# Mitochondrial cristae: lung cancer metabolism architects

**DOI:** 10.1093/lifemeta/load015

**Published:** 2023-05-12

**Authors:** Masafumi Noguchi, Luca Scorrano

**Affiliations:** Department of Biology, University of Padua, 35121 Padua, Italy; Veneto Institute of Molecular Medicine, 35129 Padua, Italy; Laboratory of Pharmacology, School of Pharmaceutical Sciences, Wakayama Medical University, 25-1 Shichibancho, 640-8156 Wakayama, Japan; Department of Biology, University of Padua, 35121 Padua, Italy; Veneto Institute of Molecular Medicine, 35129 Padua, Italy

**In a recent study published in *Nature*, Shackelford and colleagues used an innovative *in vivo* mitochondrial morphology and bioenergetics analysis pipeline to correlate the metabolic signature of non-small cell lung cancer subtypes to the ultrastructure of mitochondria and their contact sites with lipid droplets. This study paves the way for diagnostic and therapeutic protocols for lung cancer based on the rationale assessment and modulation of mitochondrial topology and ultrastructure.**

**Figure 1:**
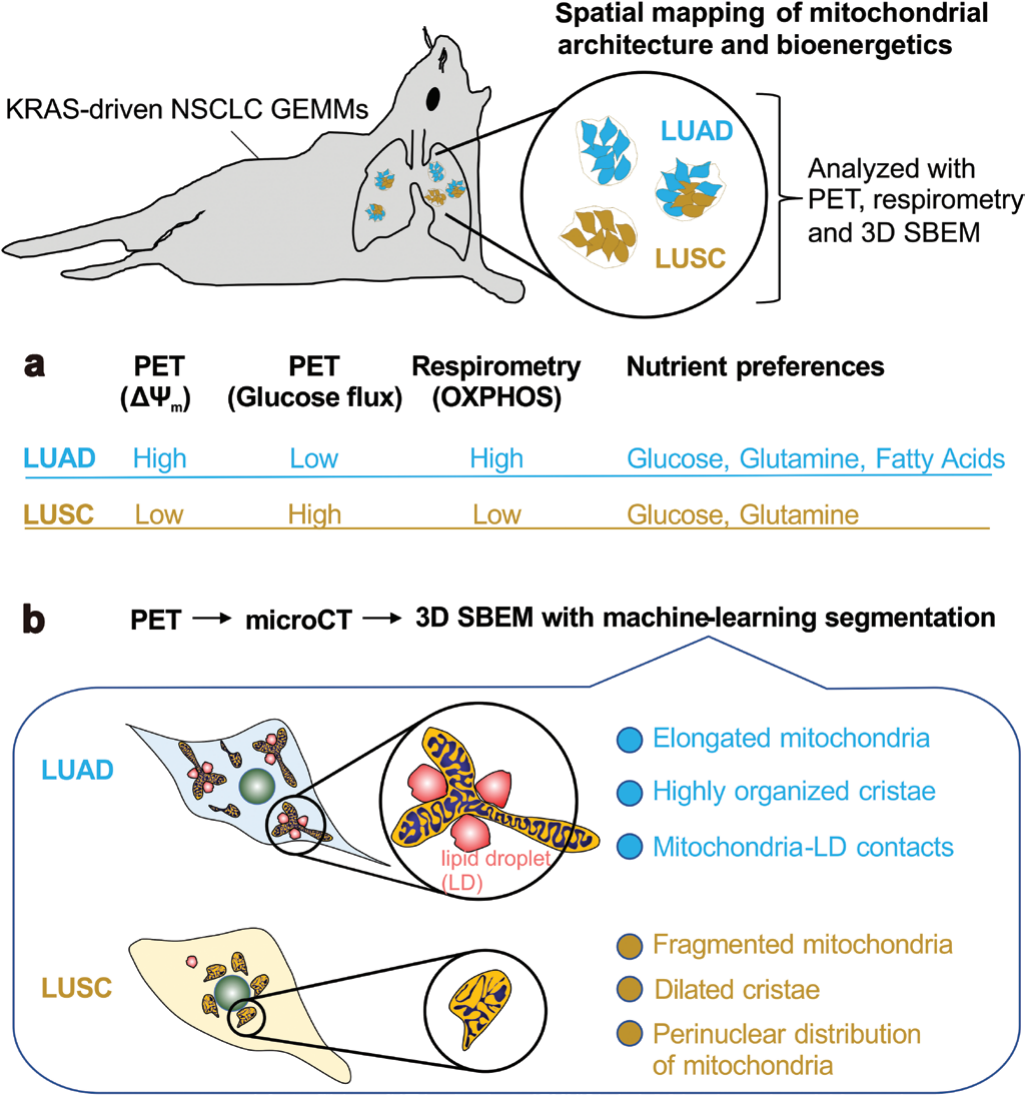
A schematic summary of the generation of heterogeneous NSCLC and spatial mapping of mitochondrial architecture and bioenergetics. (a) Differential mitochondrial bioenergetics and nutrient preferences in the tumor subtypes of NSCLC. (b) Differential distribution of mitochondria and their morphology in the tumor subtypes of NSCLC.

Mitochondria are dynamic bioenergetic structures adapting their morphology to the varying cellular pathophysiological conditions. Given the crucial role of mitochondrial morphology in the regulation of the multiple cellular processes controlled by mitochondria, it is not surprising that mitochondrial dynamics has become an important facet in cancer biology and a potential therapeutic space for different tumor types. However, our understanding of the precise role of mitochondrial dynamics in cancer has been so far mostly correlative, because of an overarching technological problem: we lack an accurate imaging method to visualize the rearrangements of mitochondrial morphology in the tumor compared to the surrounding normal tissue. Han *et al*. solved this issue by developing a method to examine *in vivo* mitochondrial structure and bioenergetics of heterogeneous cancer subtypes in non-small cell lung cancer (NSCLC). They achieved topographical mapping of mitochondria in NSCLC cellular landscape through an integrated platform that combined positron emission tomography imaging (PET), respirometry, and three-dimensional scanning block-face electron microscopy (3D SBEM) [1] (Fig. 1).

Lung cancer is the main cause of cancer-related deaths globally [2, 3]. More than 85% of these deadly lung cancers are classified as NSCLC. NSCLCs are further subdivided into two main histological subtypes: lung adenocarcinoma (LUAD) and lung squamous cell carcinoma (LUSC) [4, 5]. In the last decade, groundbreaking innovations like next-generation sequencing, the generation of genetically engineered mouse models like KRAS-driven NSCLC models, and comprehensive databases of human tumor molecular profiles have transformed our understanding of NSCLC. By moving beyond the limitations of the histopathological analysis, we are now able to scrutinize NSCLC with exceptional accuracy, delving into its molecular, genetic, and even individual cellular heterogeneity [6]. Han *et al*., building on these advancements, achieved a new milestone by developing a workflow to bioenergetically map the heterogeneous NSCLC at super resolution.

Previously, the group established a method to measure mitochondrial membrane potential (Δψ_m_) in NSCLC *in vivo* using a PET radiotracer of ^18^F-BnTP. ^18^F-BnTP can easily pass through cellular and mitochondrial membranes, and its equilibrium concentrations on both sides of the mitochondrial membrane follow the Nernst equation [7]. They found that LUAD displayed higher ^18^F-BnTP avidity than LUSC, suggesting differences in Δψ_m_ between the two histotypes. Additionally, the glucose flux, as measured by [^18^F]-FDG uptake, was inversely correlated with the ^18^F-BnTP measured Δψ_m_.

In this paper, the authors aimed at using their PET pipeline to understand whether this approach could inform us about the oxidative phosphorylation (OXPHOS) signature of the different NSCLC subtypes. To this end, they performed *ex vivo* respirometry on snap-frozen tissues to measure the maximal respiratory capacity (MRC) of mitochondria and correlate it with the *in vivo* measured ^18^F-BnTP. They found that ^18^F-BnTP uptake and MRC were directly correlated in tumor subtypes, i.e., MRC and Δψ_m_ were both lower in LUSC compared to LUAD. After PET, the researchers dissected the tumors and performed micro-computed tomography (microCT) and ultra-high-resolution 3D SBEM. MicroCT identified suitable regions for 3D SBEM analysis and allowed them to bridge the gap in the resolution between whole-tumor imaging with PET and ultrastructural analysis with 3D SBEM. Furthermore, the authors analyzed 3D mitochondrial structure by creating a custom deep-learning convolutional neural network (github.com/tiard/mito-networks-3d). Researchers could accurately quantify 20,000–50,000 mitochondrial structures in each tumor section using this advanced machine-learning-driven segmentation tool. Additionally, they determined the spatial relationships between the nucleus, lipid droplets, and mitochondria, achieving a comprehensive mitochondrial mapping in the region of interest for NSCLCs. Moreover, they could visualize the various subtypes of cristae structures within individual mitochondria in NSCLCs.

With this workflow, the authors discovered that mitochondria within the low MRC LUSC cells were smaller and more fragmented than the ones in the high MRC LUAD cells. While LUAD cells exhibited a broad spatial distribution of mitochondrial networks across the cytoplasm, the mitochondria in LUSC cells were predominantly concentrated at perinuclear regions. Additionally, 3D SBEM enabled them to visualize the ultrastructure of cristae, the structural organization of which controls respiratory chain supercomplex assembly and mitochondrial respiratory efficiency [8]. The researchers delineated the morphological classification of mitochondrial cristae within NSCLC into three distinct categories: Type I—characterized by highly organized, orthodox, or lamellar cristae structures; Type II—typified by sparse and disorganized cristae formations; and Type III—exhibiting condensed cristae configurations. While LUAD cells exhibited a mixture of Types I, II, and III cristae, mitochondria in LUSC cells were predominantly characterized by Type III cristae with fewer Type I cristae. This finding mirrored the lower basal oxygen consumption rate in LUSC versus LUAD cells. In sum, their ultrastructural imaging showed that LUSC cells consistently lacked organized Type I cristae compared to LUAD cells. By inspecting the inter-organelle interactions in the 3D SBEM images of NSCLC, the authors offered a potential explanation linking fuel source, intracellular metabolic compartmentalization to the ultrastructural organization of the different NSCLC types. They observed that LUAD cells contained several lipid droplets that were conversely absent in LUSC cells. In LUAD, mitochondria partake in contact with single or clustered lipid droplets, appearing as peri-droplet mitochondria (PDM). Notably, these PDM exhibited orthodox Type I cristae, aligned vertically at the mitochondria-lipid contact sites, suggesting that the respiratory active mitochondria of LUAD selectively interact with lipid droplets. Interestingly, the two cancer subtypes also displayed distinct fuel requirements. PDM-rich LUAD cells relied on a broad source of nutrients (glucose, glutamine, or free fatty acids) to support OXPHOS and growth. In contrast, LUSC depended on glucose and glutamine, less on fatty acid oxidation. Metabolism played a crucial role also in determining the perinuclear distribution of mitochondria in LUSC. Indeed, inhibition of glucose flux and of the related hexosamine-O-GlcNAcylation pathway led to the redistribution of mitochondria from the perinuclear region. This change was accompanied by an increase in mitochondria with Type I cristae and with OXPHOS upregulation. In essence, high glucose flux shapes mitochondrial bioenergetics and architecture, thereby defining the distinct features of LUSC.

Han *et al.* show that a refined workflow for analyzing mitochondria in NSCLC enables a new perspective on cellular mitochondrial heterogeneity and complements the classification of heterogeneous NSCLC *in vivo*. This approach not only has far-reaching implications for our understanding of basic mitochondrial biology, metabolism compartmentalization, and tumor biology, but can also advance the development of mitochondrial dynamics therapies for NSCLC. For example, their finding that cristae are heterogeneous and can shape bioenergetics in NSCLC points to these structures as potential targets in lung cancer. Drugs that specifically target the master cristae shaping factor Optic Atrophy 1 [9] exist, pointing to the possibility to use them in NSCLC patients accurately stratified for their mitochondrial subtype.

The pipeline by Han *et al*. can also help clarify how the tumor microenvironment influences mitochondrial biology *in vivo* in lung cancer cells. Tumor microenvironment appears crucial for the development and progression of lung cancer, but its recapitulation *ex vivo* is still imprecise, especially vis à vis the difficulties in accurately duplicating tumor metabolism. The *in vivo* approach devised by Han *et al*. conversely offers a refined tool to elucidate how the microenvironment influences the mitochondrial status of cancer cells. For example, factors such as differential nutrient supply provided by tumor angiogenesis [10] and the pro-tumorigenic niche created by residual macrophages [11] might affect mitochondrial architecture and bioenergetics *in vivo*. By expanding this pipeline to a dynamic analysis of NSCLC in relationship with its microenvironment, we expect to increase our understanding of the role of mitochondrial ultrastructure to unprecedented aspects of the pathobiology of cancer.
